# Diabetes and coronavirus (SARS-CoV-2): Molecular mechanism of Metformin intervention and the scientific basis of drug repurposing

**DOI:** 10.1371/journal.ppat.1009634

**Published:** 2021-06-22

**Authors:** Elizabeth Varghese, Samson Mathews Samuel, Alena Liskova, Peter Kubatka, Dietrich Büsselberg

**Affiliations:** 1 Department of Physiology and Biophysics, Weill Cornell Medicine-Qatar, Education City, Qatar Foundation, Doha, Qatar; 2 Department of Obstetrics and Gynecology, Jessenius Faculty of Medicine, Comenius University in Bratislava, Martin, Slovakia; 3 Department of Medical Biology, Jessenius Faculty of Medicine, Comenius University in Bratislava, Martin, Slovakia; University of Alberta, CANADA

## Abstract

Coronavirus Disease 2019 (COVID-19), caused by a new strain of coronavirus called Severe Acute Respiratory Syndrome Coronavirus 2 (SARS-CoV-2), was declared a pandemic by WHO on March 11, 2020. Soon after its emergence in late December 2019, it was noticed that diabetic individuals were at an increased risk of COVID-19–associated complications, ICU admissions, and mortality. Maintaining proper blood glucose levels using insulin and/or other oral antidiabetic drugs (such as Metformin) reduced the detrimental effects of COVID-19. Interestingly, in diabetic COVID-19 patients, while insulin administration was associated with adverse outcomes, Metformin treatment was correlated with a significant reduction in disease severity and mortality rates among affected individuals. Metformin was extensively studied for its antioxidant, anti-inflammatory, immunomodulatory, and antiviral capabilities that would explain its ability to confer cardiopulmonary and vascular protection in COVID-19. Here, we describe the various possible molecular mechanisms that contribute to Metformin therapy’s beneficial effects and lay out the scientific basis of repurposing Metformin for use in COVID-19 patients.

## Introduction

In late December 2019, the first case of a novel coronavirus that caused severe acute respiratory distress emerged from Wuhan, Hubei Province, China [1]. The Coronavirus Disease 2019 (COVID-19), within the next few weeks, was linked to the Severe Acute Respiratory Syndrome Coronavirus 2 (SARS-CoV-2), which targeted the respiratory tract, caused pneumonia, Acute Respiratory Distress Syndrome (ARDS), and death in severely affected individuals. The SARS-CoV-2–caused pandemic has rampantly spread across all countries, and, currently, has infected over 162 million people and is responsible for over 3.3 million deaths (as of May 16, 2021) [2].

**Fig 1:**
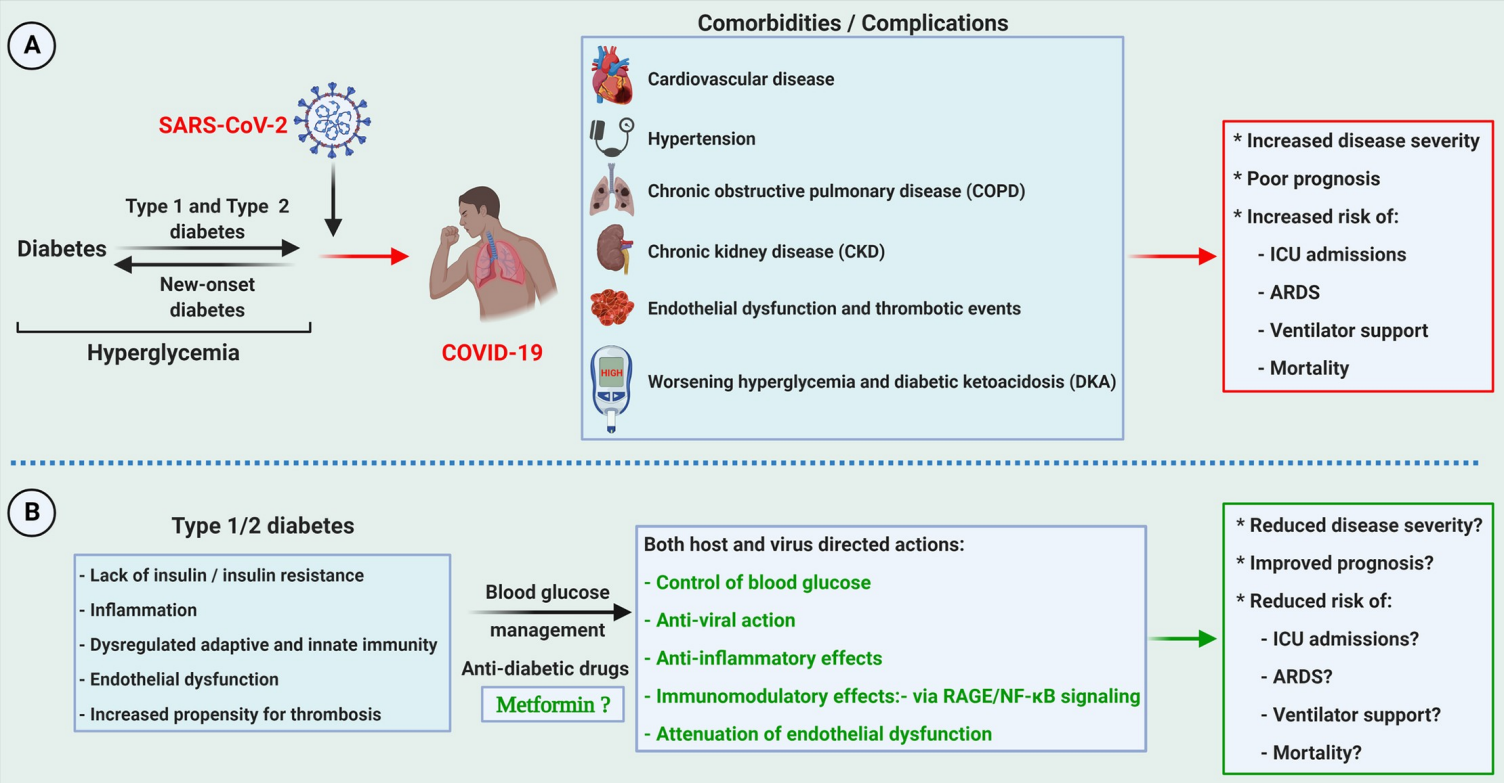
Bidirectional relationship of diabetes vs. COVID-19 and the potential benefits of the antidiabetic drug Metformin. **(A)** The relation between COVID-19 and diabetes is two sided. COVID-19 in preexisting diabetes can aggravate the disease in the presence of comorbid conditions leading to a worse prognosis. Meanwhile, new-onset diabetes, reported in patients with COVID-19 with no prior history of diabetes, could be due to adverse SARS-CoV-2 infection–mediated immune reaction and the destruction of the pancreas’ β-cells. (**B)** Proper blood glucose management can mitigate the COVID-19–related complications. Metformin has multiple pleotropic actions, including immune modulation, antiviral, and anti-inflammatory effects that could mitigate disease prognosis in diabetic COVID-19 patients. Repurposing drugs through a systematic approach reduces the need for clinical testing and risk assessment and helps identify off-target effects or newly identified targets. Created with BioRender.com. ARDS, Acute Respiratory Distress Syndrome; COVID-19, Coronavirus Disease 2019; SARS-CoV-2, Severe Acute Respiratory Syndrome Coronavirus 2.

Zoonotic transmission of coronaviruses have caused diseases in humans, such as Severe Acute Respiratory Syndrome (SARS, in 2003), Middle East Respiratory Syndrome (MERS, in 2012), and most recently, the COVID-19 [3]. The COVID-19 spreads through respiratory droplets and typically has an incubation period of 7 to 14 days [3]. While symptoms and severity varied from patient to patient, common symptoms associated with SARS-CoV-2 infections were fever, cough, sore throat, and difficulty breathing. A more severe outcome with higher mortality rates among COVID-19 patients was associated with age and risk factors such as hypertension, diabetes, obesity, cardiovascular disease, and chronic kidney diseases (Fig 1A) [4,5]. Molecular level studies on viral pathogenesis identify the binding of SARS-CoV-2 to the angiotensin converting enzyme 2 (ACE2) receptor as an essential step initiating the pathogenesis of COVID-19. The viral spike protein binding to the host ACE2 receptor reduces ACE2 availability and disrupts its control over the renin–angiotensin–aldosterone system (RAAS) [6,7]. In turn, this initiates the inflammatory response via the NF-κB and JAK/STAT signaling–mediated gene transcription and translation of the pro-inflammatory cytokines (cytokine storm), contributing to increased disease severity and mortality among affected individuals [6,7].

Although diabetes had no significant link to susceptibility to SARS-CoV-2 infection, it significantly correlated with disease severity, the occurrence of ARDS, ICU admissions, and mortality [8,9]. Other noticeable observations from China, Italy, and the United Kingdom suggest an increased incidence of new-onset type 1 diabetes in children and youth below 25 years [10,11]. High mortality rates in COVID-19 were reported in patients with poor blood glucose management compared to patients with well-controlled blood glucose levels [3]. Blood glucose levels within the range 3.9 to 10.0 mmol/L reported a lower mortality rate than in patients with blood glucose levels above 10.0 mmol/L. The glycemic characteristics predict the clinical outcome showing a more extended hospital stay and a higher mortality rate with high blood glucose levels [12]. Clinical evidence indicated that better blood glucose management results in an improved outcome in patients with multiple organ injury and reduced the mortality rate in patients with COVID-19 [13,14].

Various glucose-lowering agents, such as insulin and Metformin, are frequently used in patients with diabetes for blood glucose management in COVID-19 patients. Metformin, also known as “Glucophage,” is one of the most prescribed drugs for managing type 2 diabetes [15–18]. The glucose-lowering effect of Metformin is attributed to the increased glucose uptake by the muscles, decreased conversion of glycogen to glucose (glycogenolysis) and reduced glucose synthesis from noncarbohydrate sources (gluconeogenesis), and decreased absorption of glucose from the intestine. Recently, research focused on additional beneficial effects of Metformin, such as its cardioprotective, vasculoprotective, antioxidant, and antitumor effects [17,19]. Its anti-inflammatory effects are appreciated in the favorable prognosis of COVID-19 [20]. Metformin’s molecular mechanisms show its involvement in pathways controlling inflammation, glucose metabolism, vascular smooth muscle function, and in viral pathogenesis (Fig 1B) [20,21]. Here, we provide an in-depth review of the various antiviral, anti-inflammatory, immunomodulatory, cardio- and vasculoprotective effects of Metformin and possible molecular mechanisms by which COVID-19 patients benefit from Metformin intervention.

### COVID-19 and diabetes

Diabetic individuals are frequently associated with a higher susceptibility to bacterial/viral infections and follow a severe disease course than their nondiabetic counterparts [22,23]. Observations from previous infections like SARS (caused by the SARS-CoV-1) identified an underlying relationship between disease course and diabetes [24]. Hyperglycemia and preexisting diabetes were identified as independent predictors of mortality and morbidity in SARS patients [24]. Similarly, studies reported an increased risk of COVID-19 severity in patients with type 2 diabetes [25] with high morbidity and mortality rate [13,20,26]. Several mechanisms behind increased severity are related to a compromised immune system, diabetes-associated endothelial dysfunction, and decreased virus clearance in diabetic COVID-19 patients [27,28].

A population-based cohort study reported a sharp rise in COVID-19–related death among the patients with diabetes (both type 1 and 2) compared to the years before the start of the pandemic [29]. Poor glycemic control in patients as indicated by HbA1c of 59 mmol/mol (7·6%) or higher strongly correlated with a higher mortality rate (hazard ratio [HR] = 2.23) in type 1 diabetes and 1.61 in type 2 diabetes) [29]. Other studies reported similar findings, such as a higher adjusted (odds ratio [OR] = 3.5) of mortality in type 1 diabetic patients after adjusting for host conditions such as age, sex, socioeconomic deprivation, ethnicity, and others [30]. An emergence of new-onset diabetes in children (80% increase) during the COVID-19 pandemic was reported [11]. Although no direct link is established between new-onset diabetes and COVID-19, data from previous studies on SARS-CoV-1 show the presence of the ACE2 receptor on the pancreatic β-cells, which facilitates viral binding and infection, leading to the destruction of the β-cells and insulin insufficiency that causes hyperglycemia [11]. Based on current clinical data, it appears that SARS-CoV-2 may trigger severe diabetic ketoacidosis (DKA) in individuals with new-onset type 1 diabetes [31]. However, up to now, there is no unambiguous evidence that SARS-CoV-2 induces type 1 diabetes [31].

SARS-CoV-2 infection can affect multi-organ systems, and this proportionally correlated with the expression and distribution pattern of ACE2 receptors in various organs. Emerging studies supported the viral tropism to both exocrine and endocrine cells of the pancreas and demonstrated modification in the pancreas’ morphological, translational, and functional aspects, ultimately impairing insulin secretion [32,33].

An elevated blood glucose level is also linked to exacerbated inflammatory response, as seen in COVID-19. Given the importance of monocytes and macrophage’s role in immune response and the predilection of the virus to infect these cells, this could explain the worsened prognosis in COVID-19. An in vitro study conducted in monocytes under increasing glucose concentrations showed elevated viral load, ACE2, and interleukin (IL)-1β expression with SARS-CoV-2 infection [34]. In parallel, unpublished reports on clinical samples confirmed SARS-CoV-2 infection in monocytes and indication of pyroptosis in monocytes isolated from COVID-19 patients [35]. Unlike the infections with other respiratory viruses, a glycolytic trait was identified in monocytes infected with SARS-CoV-2. These results were further confirmed with 2-deoxy-D-glucose (2-DG; inhibits glucose flux) by decreasing viral load in monocytes and decreased expression of tumor necrosis factor (TNF)α, IL-6, α, β, and λ interferon (IFN) [34]. A study on COVID-19 patients confirmed these preclinical findings. Clinical data revealed that elevated glucose levels increased cytokine profiles and immune response in patients. Compared to nondiabetic COVID-19 patients, diabetic COVID-19 patients are characterized by a higher percentage of CD4^+^ T cell and a lower percentage of CD8^+^ T cells and higher serum levels of IL-6, IL-2, IL-10, and INFγ. Moreover, serum levels of TNFα, IL-4, IL-2, IL-10, and INFγ were significantly higher in the diabetic group than the impaired fasting glucose group [36]. Whether the typical “cytokine storm” immune response pattern often indicated in COVID-19 is due to viral destruction of immune cells or is due to preexisting underlying conditions in diabetes needs to be distinguished as this would change the approach to treatment strategies.

**Fig 2:**
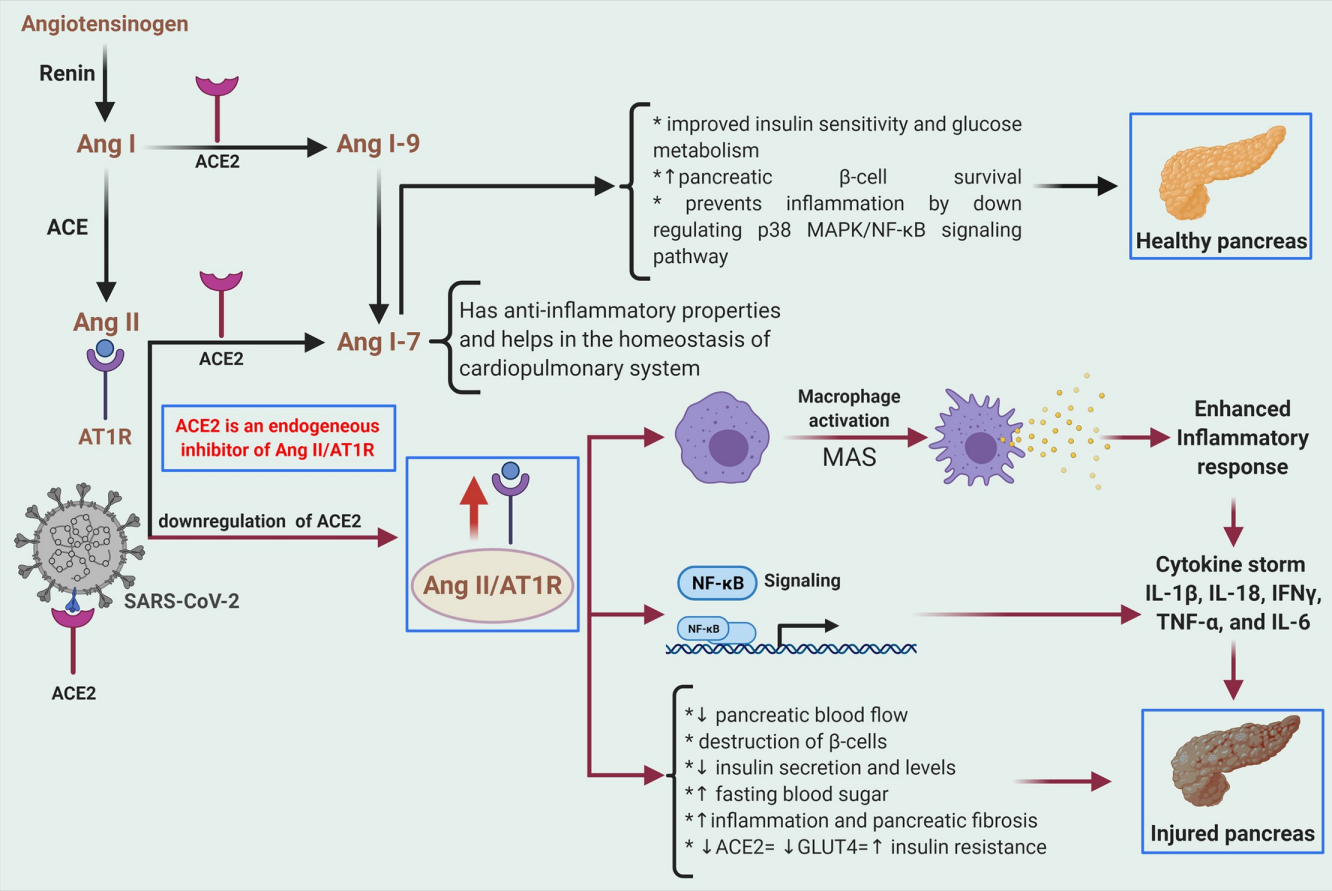
RAAS/SARS-CoV-2 axis in exaggerated immune response and acute pancreatic injury. RAAS is an essential player in immune response, glucose metabolism, and regulating blood pressure. In the RAAS system, Renin, a proteolytic enzyme, converts angiotensinogen to AngI, and, further, the enzyme ACE converts AngI to AngII. Subsequently, the membrane-bound ACE2 metabolizes AngII to Ang 1–7, which has beneficial metabolic effects. The RAAS cascade continues with the binding of AngII to AT1R or AT2R and exerts various pathophysiological effects. Under normal physiological conditions, the AngII/AT1R axis is kept under check by ACE2/Ang 1–7 axis. Down-regulation of ACE2 in SARS-CoV-2 infection weakens the ACE2/AngI/MAS axis’s inhibitory effect over ACE2/AngII/AT1R axis, which will result in the pro-inflammatory response and subsequent tissue/organ damage. Proper activation and control of RAAS system are essential for the cardiovascular and pulmonary systems’ proper functioning. It also has a significant role in the pathophysiology of insulin resistance. Increased activity of AngII can alter insulin signaling, insulin secretion, and insulin sensitivity. Created with BioRender.com. ACE2, angiotensin converting enzyme 2; IFN, interferon; IL, interleukin; RAAS, renin–angiotensin–aldosterone system; SARS-CoV-2, Severe Acute Respiratory Syndrome Coronavirus 2; TNF, tumor necrosis factor.

It is essential to relate the RAAS system to the pathophysiology of COVID-19–related diabetic complications. ACE2, a zinc-dependent transmembrane metalloproteinase and a potent receptor for SARS-CoV-2 binding, is part of the RAAS signaling and plays a protective role in regulating the normal functioning of the cardiovascular and the pulmonary system [37]. The components, signaling of the RAAS cascade, and the beneficial role of ACE2 in balancing ACE2/Ang 1–7/MAS axis by down-regulating the harmful Ang II/AT1R axis are depicted in (Fig 2) [37,38].

Retrospectively, in COVID-19, binding of the spike protein of SARS-CoV-2 to ACE2 receptor down-regulates its activity and leads to the accumulation of Ang II, and the overactivation of angiotensin AngII/AT1R axis trigger macrophage activation and activation of NF-κB signaling, leading to release of several inflammatory cytokines affecting multi-organ functions (Fig 2) [38].

Consistently, in the pancreas, a dysregulated RAAS is associated with vascular damage, inflammation, and decreased GLUT4 translocation by interfering with PI3K/AKT, MAPK, and NF-κB pathway [37].

The vulnerability of β-cells to SARS-CoV-2 was supported by ex vivo studies, which demonstrated replicating viral particles in pancreatic cells with robust expression of viral proteins such as viral spike (S) and nucleocapsid (N) protein and the co-expression of 2 essential docking proteins ACE2 and TMPRSS2 for viral entry [32]. These results were confirmed by blocking the expression of viral proteins with 5-μM remdesivir [32]. Transcriptional studies in human pancreatic islets infected with SARS-CoV-2 indicated the up-regulation of several genes linked to IFN-stimulated genes and down-regulation of genes linked to β-cell function [32].

Another feature of COVID-19 that increased patients’ risk with diabetes is the diabetes-associated endothelial dysfunction, characterized by hypercoagulation, higher incidence of thrombotic events, and microvascular complications [39–46]. Interestingly, studies show that SARS-CoV-2 can gain entry into endothelial cells via the endothelial cell surface ACE2 receptors [43,47]. Another study reported a different mechanism for impaired endothelial function. Pericytes were identified as a port of viral entry rather than endothelial cells contributing to a leaky endothelial barrier [48]. SARS-CoV-2 particles and host inflammatory cells were present within the endothelial cells, indicative of impaired integrity of the vasculature and was related to more ICU admissions among COVID-19 patients [43,47]. These patients presented with higher levels of D-dimer, von Willebrand factor (VWF) and soluble P-selectin, and factor VIII activity and manifested venous thromboembolism and microvascular lung thrombosis [44–46]. Cumulative evidence suggests that a glycolytic trait can influence the course of the disease by promoting viral tropism and negatively modulate the immune response and functional integrity of tissues, including endothelium.

## Metformin intervention in COVID-19

### Clinical observations

As mentioned, diabetic COVID-19 patients followed a more severe disease course and had higher ICU admissions and mortality rates than their nondiabetic counterparts [4]. Overall, maintaining blood glucose levels within the normal range improved prognosis and survival in diabetic COVID-19 patients [13]. However, the choice of glucose-lowering agent used to manage or achieve normal blood glucose levels may prove critical while treating diabetic COVID-19 patients, especially when other clinical factors, comorbidities, and drugs required to treat other symptoms should be taken into account.

It was reported that insulin decreased disease severity and mortality rates in diabetic COVID-19 patients, possibly due to its anti-inflammatory and immunomodulatory effects and the reduction of blood glucose levels in the patients [49,50]. However, other reports claim that insulin administration in COVID-19 patients worsened the clinical profile and was correlated with poor prognosis possibly due to the insulin-mediated inhibition of disintegrin and metalloproteinase domain–containing protein 17 (ADAM17), thereby facilitating the proteolytic cleavage and shedding of the active ecto-domain of ACE2 and thus increasing the availability and activity of ACE2 for SARS-CoV-2 infection [51,52]. On the contrary, reports indicate several benefits of using Metformin for COVID-19 therapy, independent of its ability to decrease insulin resistance and improve glucose utilization, thus reducing glucose levels in circulation [20,53]. With the limited information available, it is indeed difficult to ascertain whether patient differences and disease severity contributes differential effects of Metformin and insulin given the fact that both reduces blood glucose levels. Hence, more studies are warranted in this regard.

Multiple studies identified diabetes as an independent risk factor associated with a higher mortality rate among diabetic COVID-19 patients [54]. The study conducted among a diverse population noted that patients with diabetes using Metformin to manage their hyperglycemia had a 3-fold reduction in mortality in the case of COVID-19 [54]. In contrast, those on an insulin treatment regimen showed no such positive effect [54]. Remarkably, the ability of Metformin to reduce mortality rate was observed after correcting for other risk factors, such as age, sex, race, obesity, and hypertension, or chronic kidney disease and heart failure that reduce survival among COVID-19 patients [54]. Several retrospective and meta-analysis studies reported a significant reduction in COVID-19-related mortality among high-risk patients with diabetes who were on a Metformin treatment to manage their blood glucose levels [55–58].

Furthermore, a retrospective cohort study among type 2 diabetic or obese (BMI at least 30 kg/m^2^) COVID-19 participants found that Metformin treatment was associated with a reduction in disease severity and mortality among women, but not in men who were hospitalized with COVID-19, indicating a gender-dependent effect [59]. Similarly, in another retrospective cohort study, Metformin was demonstrated to be potentially effective in reducing ARDS incidence in COVID-19 patients with type 2 diabetes, especially females [21,60]. It was noted that, while Metformin did not increase mortality due to acidosis, a high dosage of Metformin could impair renal function and increase the risk of acidosis among 2 diabetic COVID-19 patients [61]. The continuation of Metformin therapy was, however, recommended, owing to its ability to reduce inflammation and heart failure, among 2 diabetic COVID-19 patients, albiet under continuous monitoring for lactic acidosis and deterioration of renal function [61].

In addition to its antihyperglycemic effect, which by itself reduces the susceptibility to different infections and reduces disease severity, Metformin exerts its beneficial effects in combating COVID-19 in multiple ways. It confers protection against several infections (including those caused by coronaviruses) through several host-directed therapeutic mechanisms [21,53,62]. Metformin is widely studied for its antiviral, anti-inflammatory, immunomodulatory, cardio- and vasculoprotective effects [62–64]. The following chapters examine the mechanistic aspects of Metformin action on virus–host-directed therapies and, retrospectively, links to prognosis in diabetic COVID-19 patients.

### Metformin’s effect on ACE2 stability and viral entry

A viral infection starts with the virus attachment to the host cell. Studies proposed different virus-specific/dependent mechanisms by which virus gains entry into/infects the host cell [65]. While some viruses fuse with the plasma membrane and then release the viral genome, others enter by endocytosis. Both membrane fusion and receptor-dependent endocytosis were reported in SARS-CoV-1 entry [66,67]. For COVID-19, while the cell surface ACE2 receptor of the respiratory tract’s target cells facilitated the SARS-CoV-2 viral binding and infection of the host cell, the transmembrane TMPRSS2 cleaved the viral spike protein and supported virion entry into the cell [68,69]. Although the viral replication is primarily confined to the respiratory tract, studies in a nonhuman primate model reveal replication of SARS-CoV-2 in extrapulmonary tissues such as ilium, colon, pancreas, and tonsils [32,70]. However, it is noteworthy that the ACE2 receptor is expressed in various organs and tissues such as the brain, heart, liver, intestine, pancreas, kidney, adipose tissue, and vasculature, making them possible targets for SARS-CoV-2 infection, which may explain the damage reported in multi-organ systems in COVID-19 patients [70–72]. Interestingly, the risk factors such as diabetes and obesity correlated with an increased expression of ACE2 in various tissues, leading to a possible increase in the viral load within these tissues [73,74]. Furthermore, the shedding of ACE2 from the cell surface and its redistribution, commonly observed in diabetic and obese individuals, supports the viral spread to different regions within the body [73].

The various stages of viral infection, starting with binding the spike protein of the SARS-CoV-2 virus to the host ACE2 receptor and leading to the release of assembled virions by exocytosis, are depicted in Fig 3① to 3⑩ and is described in several reviews [75,76].

**Fig 3:**
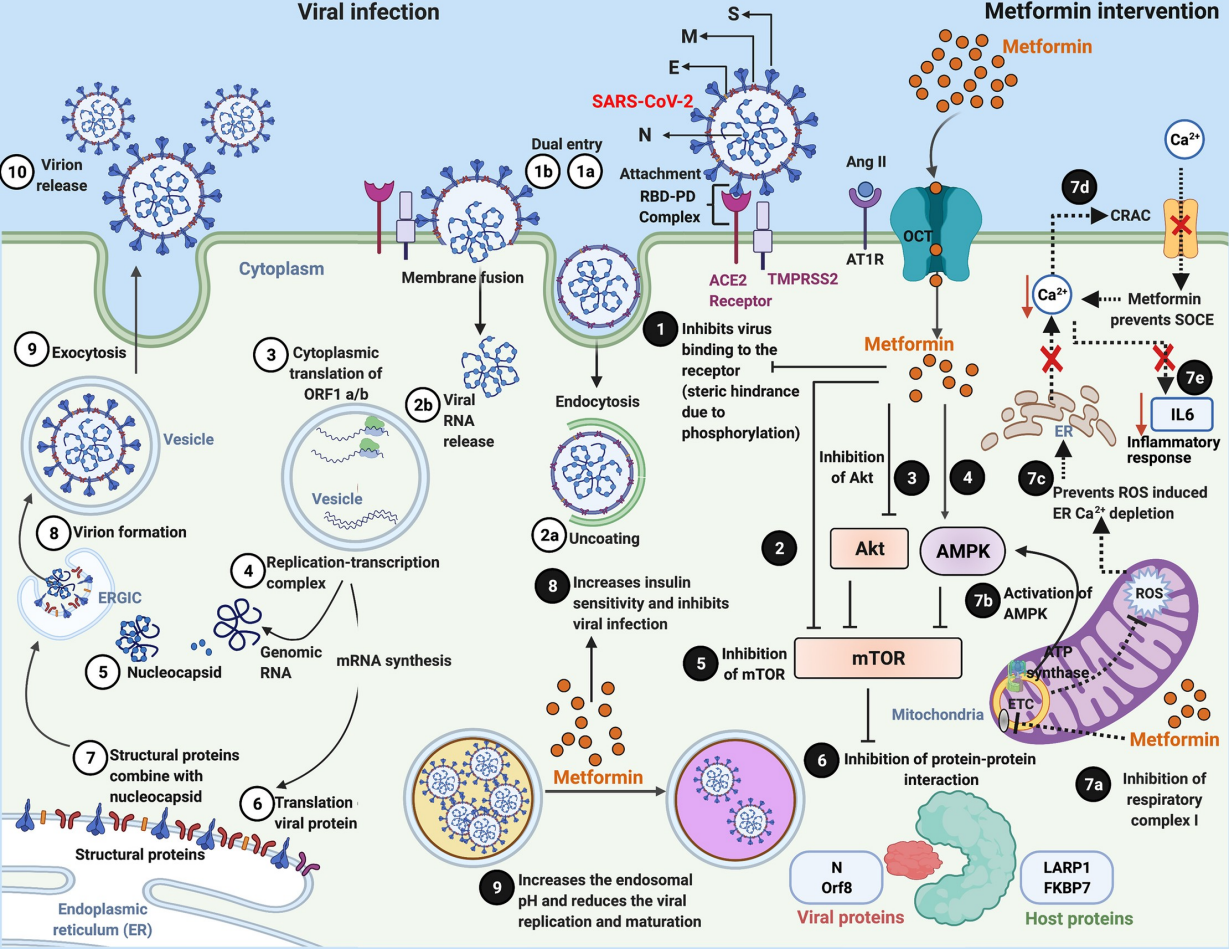
Viral pathogenesis and Metformin’s host- and virus-directed interventions in the attenuation of the disease process. Numerical indicators ①–⑩ illustrate the different stages of a typical viral infection, replication, transcription, protein translation, virion assembly, and release. Viral infection starts with binding of the spike [S] protein of the virus to the host receptor ACE2 to form the RBD–PD complex. This attachment is primed by another membrane protein called TMPRSS2 “①a.” “①b” indicates another form of viral entry through host membrane fusion. Viral entry by endocytosis is followed by uncoating “②a” and release of the viral RNA genome “②b,” which is translated into viral RNA polymerase proteins resulting in the formation of ③ sub-genomic (−) RNAs then used as a template from sub-genomic (+) mRNAs. After that, the following processes occur: the cytoplasmic viral RNA replication ④ and nucleocapsid [N] protein transcription ⑤ and subsequent translation ⑥ of structural viral proteins [S], membrane [M], and envelope [E] protein in the ER; followed by assembly of structural [S], [M], and [E] protein with the [N] protein viral genome RNA complex ⑦ and mature virion assembly ⑧ in the ERGIC. The assembled virions bud off from the Golgi vesicle ⑨ and are released ⑩ by exocytosis. Numerical indicators ❶–❾ illustrate the potential sites of virus–host-directed action of Metformin. ❶ Metformin can potentially inhibit the virus’s attachment to the host plasma membrane by blocking the virus from binding to the host receptor ACE2. Metformin directly ❷, indirectly via Akt inhibition ❸ or via AMPK activation ❹ inhibits mTOR activity ❺ resulting in the suppression of virus–host protein interaction ❻. Inhibition of respiratory complex I by Metformin “❼a” activates AMPK “❼b” and results in inhibiting the mTOR signaling. Metformin also inhibits mitochondrial generation of ROS “❼c,” which subsequently prevents ROS-induced ER calcium (Ca^2+^) depletion and suppresses the activation of CRAC channel “❼d,” preventing SOCE ultimately preventing a rise in intracellular Ca^2+^ and subsequent release of the inflammatory mediator IL-6 “❼e,” which is often associated with COVID-19 and mediates thrombosis. Metformin increases insulin sensitivity ❽ and inhibits viral infections, modifies endosomal pH, and reduces viral ❾ replication and maturation. Created with BioRender.com. ACE2, angiotensin converting enzyme 2; COVID-19, Coronavirus Disease 2019; CRAC, Ca^2+^ release-activated Ca^2+^ channel; ER, endoplasmic reticulum; ERGIC, ER–Golgi intermediate compartment; IL, interleukin; PD, peptidase domain; RBD, receptor-binding domain; ROS, reactive oxygen species; SARS-CoV-2, Severe Acute Respiratory Syndrome Coronavirus 2; SOCE, store-operated calcium entry.

Metformin, a direct activator of AMPK, is known to promote ACE2 phosphorylation (Ser 680), causing a conformational change, and, therefore, in theory, a steric hindrance would prevent viral binding to the host ACE2 receptor (Fig 3-❶) [53]. Moreover, Metformin-mediated AMPK-dependent phosphorylation of ACE2 (which rescues it from poly-ubiquitination and subsequent 26S proteasomal degradation) extends ACE2 half-life and thus potentially offers lung protection. In addition, changes in 3D conformation of ACE2 extracellular domain induced by posttranslational modifications could potentially decrease SARS-CoV-2 viral recognition [77]. On the contrary, it is hypothesized that Metformin would stabilize ACE2 expression in the respiratory tract and possibly increase SARS-CoV-2 infection [78]. However, it is noteworthy that viral binding to the ACE2 reportedly decreases ACE2 stability and availability, leading to an imbalance in the RAAS and the manifestation of harmful biological effects (vasoconstrictive, hypertrophic, fibrotic, proliferative, and pro-inflammatory effects and induction of oxidative stress) in different organ systems [38,71]. Due to the role in the activation of AMPK and further downstream ACE2, Metformin can prevent ACE2 down-regulation mediated by SARS-CoV-2 [79]. The Metformin-mediated (1) increase in the levels of ACE2 and (2) the phosphorylation of ACE2 subsequently regulates RAAS offering cardiopulmonary protection, stability to the pulmonary endothelium, and mitigates pulmonary hypertension [53,80]. Specifically, Metformin treatment–associated ACE2 expression and stability can positively modulate the beneficial arm (ACE2/Ang 1–7) of the RAAS and prevent pancreatic damage and new-onset diabetes in COVID-19 patients by protecting and maintaining the normal function of the pancreas [81].

### Metformin-mediated mTOR inhibition

A recent study revealed a reduction in mortality among SARS-CoV-2–infected diabetic nursing home residents who were on Metformin to manage their blood glucose levels, and this was correlated with potential mTOR inhibitory effects of Metformin [82]. Mechanistically, Metformin may disrupt the interaction between the host and viral proteins necessary for viral replication, virion assembly, and pathogenesis [15,16,53]. Metformin treatment (1) directly (Fig 3-❷), (2) via the inhibition of AKT (Fig 3-❸) and/or (3) activation of AMPK (Fig 3-❹) inhibits the mTOR signaling pathway (Fig 3-❺), thus halting the cellular translational process, which is necessary for the synthesis of host and viral proteins. Alternatively, Metformin treatment–mediated inhibition of mTOR via REDD1 and phosphorylation of Raptor has also been reported [15,19]. Gordon and colleagues identified 332 high-confidence human protein–viral (SARS-CoV-2) protein interactions, out of which 66 protein interactions could act as targets for certain drugs, such as Metformin, that the FDA has already approved [83]. Interestingly, 2 hosts (human) proteins, LARP1 and FKBP7, which are regulated by the mTOR signaling pathway, interact with the viral (SARS-COV-2) N and Orf8 proteins, respectively (Fig 3-❻) [53]. NSP7, M viral proteins, and NDUF host protein of the electron transport chain are Metformin targets [83]. Thus, the host–viral interaction opens up the avenue of co-therapies, repurposing of drugs, and identification of new drug targets.

### Metformin-mediated immunomodulatory and anti-inflammatory effects

In COVID-19 patients, SARS-CoV-2 binding to its ACE2 receptor and decrease in ACE2 availability creates an imbalance in the RAAS, resulting in the hyper-activation of AngII/AT1R axis and triggering the NF-κB activation mediated inflammatory process and synthesis and secretion of pro-inflammatory cytokines (TNFα, IL-6, IL-1, and IL-1β), which, in part, explains the severe disease manifestations, multi-organ failure, and higher mortality in diabetic COVID-19 patients [38,84]. Of particular interest is the ability of the SARS-CoV-2 virus to infect and damage the endothelial cells causing endothelial dysfunction, owing to the presence of endothelial ACE2 receptors [43,45,47]. A higher incidence of endothelial dysfunction, hypercoagulation, higher incidence of thrombotic events, and microvascular complications was observed in COVID-19 [39–46]. Interestingly, endothelial dysfunction (mediated by diabetes-induced oxidative stress and reduced nitric oxide levels) and the existence of a prothrombotic state are hallmarks of overt diabetes and possibly may exacerbate COVID-19–related vascular complications [8,85]. Furthermore, during diabetes, the activation of receptors of advanced glycation end products (RAGE) by AGE and other ligands that trigger RAGE mediate the transcription of NF-κB–dependent pro-inflammatory and cell adhesion molecule coding genes that contribute to endothelial dysfunction and chronic vascular complications and coagulation supported by the increase in vascular hyper-permeability, increased leukocyte adhesion, and extravasation in diabetes [86–89].

Although Metformin has multiple targets, the inhibition of complex 1 of the mitochondrial electron transport chain is the most established mechanism related to (Fig 3-❼a), increasing the AMP/ATP ratio, tipping the balance toward AMPK activation (Fig 3-❼b), and subsequent mTOR inhibition. The mitochondrial electron chain inhibition also suppresses reactive oxygen species (ROS)-induced oxidative stress (Fig 3-❼c) and attenuates endothelial dysfunction and senescence. Exacerbated ROS mediate the release of IL-6 (a pro-inflammatory cytokine linked to increased disease severity and mortality in diabetic COVID-19 patients) via the accumulation of intracellular Ca^2+^ due to the opening of Ca^2+^ release-activated Ca^2+^ (CRAC) channels [90,91]. Metformin treatment–mediated reduction of ROS, in turn, prevented the depletion and release of Ca^2+^ from the endoplasmic reticulum and inhibited Ca^2+^ entry via CRAC (Fig 3-❼d and 3-❼e), thereby preventing Ca^2+^-mediated IL-6 release [91].

The immunomodulatory effects of Metformin (as evidenced by the inhibition of monocyte–macrophage differentiation, suppression of the pro-inflammatory capacity of activated macrophages, and the differentiation of T cells into regulatory and memory T cells) is linked to the Metformin treatment–associated activation of AMPK, subsequent mTOR inhibition, and reduction of oxidative stress [92]. Furthermore, Metformin reportedly inhibits RAGE-mediated NF-κB activation and subsequent up-regulation of genes that code for several pro-inflammatory cytokines and cell adhesion molecules in vascular endothelial cells, smooth muscle cells, and macrophages, thus dampening the inflammatory and immune response and thus conferring vascular protection [93–97].

### Metformin and reduced insulin resistance: A possible role in the inhibition of virus infection

The reduction of blood glucose and increase in insulin sensitivity significantly reduces the susceptibility to viral infections and disease severity among affected individuals (Fig 3-❽) [98,99]. Metformin maintains pancreatic homeostasis, owing to its beneficial effects on β-cells by reducing insulin resistance, increasing viability of β-cells, and promoting glucose metabolism [98,100].

Metformin actions on cellular mechanism involve AMPK activation and translocation of GLUT1/4 glucose transporters to the plasma membrane, facilitating enhanced glucose uptake by cells [15,98]. Insulin signaling has a prominent role in boosting the immune system and fighting against infections. The central immune cells such as T cells, B cells, or macrophages express insulin receptors (IRs) [101]. The ligand binding to IR triggers a cascade of signaling transduced through PI3K/AKT/mTOR pathway [101]. Poor T-cell function correlates with low viral clearance and inadequate vaccine response, often associated with impaired insulin signaling [101].

ACE2 plays a crucial role in maintaining glucose homeostasis via the activation of Ang (1–7)/MAS receptor axis, which, in turn, increases the survival of β-cells of the pancreas and maintains insulin secretion [102]. Conversely, data support that an altered function of ACE2 or an altered local RAAS system favors the onset of type 2 diabetes [102]. Additionally, the protective role of ACE2 against insulin resistance is enhanced by Ang (1–7) levels via the expression of GLUT4 and the transcription factor myocyte enhancer factor 2A (MEF2A) [103]. In summary, ACE2 is a potential target for therapeutic intervention where Metformin can block the activated AngII/AT1R/insulin signaling pathway and the associated immune response.

### Metformin alters endosomal pH and virus survival

Viruses use various escape mechanisms to evade host surveillance and gain entry into the host cell. The mode of viral entry depends upon the host cell type, and the endocytic route of SARS-CoV-2 in primary lung epithelial cells was established. Wang and colleagues reported a clathrin- and caveolae-independent endocytic mechanism for SARS-CoV-1 [104]. This endocytosis process was pH- and receptor-dependent and involved the binding of viral spike protein with the ACE2 receptor, followed by the internalization of the virus and the ACE2 receptor into the endosome and recycling of the ACE2 receptors back to the surface of the cell surface membrane [104]. Endosomal pH is a crucial factor for virus survival within the host cell. A low intracellular pH may favor SARS-CoV-2 binding to the host cell, its multiplication, and endosomal virion maturation [105]. Hence, targeting endosomes for therapeutic intervention using drugs capable of altering the endosomal pH may inhibit viral maturation, assembly, and survival within the host cell. Considering the different modes of viral entry of SARS CoV-2, targeting endosomal pH is significant in the endocytic mode of viral replication but not necessarily in the membrane fusion mode of viral entry. The vacuolar ATPase (V-ATPase) and endosomal Na^+^/H^+^ exchangers (eNHEs) as the primary regulators for endosomal pH are known targets for Metformin. Hence, it is possible that Metformin intervention increases the cellular and endosomal pH and suppresses the endocytic cycle and virion maturation (Fig 3-❾) [106,107].

### Metformin on gut microbiota

Metabolic syndrome like diabetes is often associated with leaky gut and increased systemic infection [108]. Apart from the involvement of the respiratory system in COVID-19, the gastrointestinal (GI) tract contributes to the manifestation of symptoms such as diarrhea, vomiting, stomach discomfort/pain, and loss of appetite during SARS-CoV-2 infection. Evidence shows that disease severity, as indicated by cytokines and inflammatory markers, is linked to gut microbiome composition, thus emphasizing the immunomodulatory role of the gut microbiome [109]. Gut microbes such as *Faecalibacterium prausnitzii*, *Eubacterium rectale*, and several bifidobacterial species known for their immunomodulatory roles were depleted in patients with COVID-19 [109]. This dysbiosis directly correlated with COVID-19 severity and persistent infection of SARS-CoV-2 in the gut epithelium [110]. Moreover, studies identify a crucial link between the gut microbiome and lung health, coined by the term “gut–lung axis,” where gut dysbiosis was associated with ARDS development [111,112].

Reports from in vivo studies demonstrated that changes in the gut microbiota influence intestinal integrity, permeability, and glucose tolerance, which may partially explain the COVID-19 progression in type 2 diabetes [113]. Furthermore, ACE2, expressed in the gastrointestinal tissue, regulates gut homeostasis and innate immune function. A review by Pollak discusses Metformin’s antidiabetic and immunomodulatory effects by influencing gut microbiota [63]. The maintenance of the gut microbiota as Metformin’s mechanism of beneficial action in COVID-19 is implicated by Metformin’s ability to change the composition of gut microbes and shift in the functional aspect of the gut microbiome as indicated by the reduction of %HbA1c and fasting blood glucose concentrations [114]. Overall, Metformin, beyond its classical action as an antidiabetic drug, reveals significant therapeutic potential against COVID-19, including its influence as an antimicrobial agent, immunomodulatory agent, and ACE2 stabilizer, and agent that regulates gut microbiota composition and maintains gut homeostasis.

Table 1 presents an overview of the potential pharmacological actions already discussed above.

**Table 1:** Overview of Metformin action and the scientific basis of drug repurposing.

Metformin effectors: Receptors, transporters, or organelles	Cellular components targeted by Metformin	Signaling	Effects	Reference(s)
Cell membrane receptor	ACE2	• AMPK-dependent phosphorylation at S680	• Inhibition of viral spike protein from binding to ACE2 due to steric hindrance • Stabilization of ACE2 and better lung protection	[80,115]
Mitochondrial electron transport chain	Inhibition of respiratory complex I	• Decreased ROS production, decreased IL6 production • DC-SIGN–dependent pathway inhibit mtDNA release and platelet activation	• Inflammatory gene expression • Decreased αIIbβ 3 expression, P-selectin, and cytosolic calcium • Prevents thrombosis	[90,91,116]
Glucose transporters	GLUT 1/4	• AMPK activation	• Enhanced glucose uptake	[98]
Macrophages Vascular endothelial cells	AMPK	• RAGE/NF-κB inhibition	• Suppression of pro-inflammatory mediators IL-8 and IL-1α	[94,117,118]
Protein–protein interaction	NDUFAF2/Nsp7 LARP, FKBP7/ N, and Orf8	• mTOR inhibition	• Inhibits virus–host protein interaction	[119]
Endosomes	Na^+^/H^+^ Exchanger, V-ATPase	• Increases intravesicular pH • Insulin signaling	• Suppression of virion assembly and maturation • Improving insulin sensitivity by targeting endocytic cycle	[106]

ACE2, angiotensin converting enzyme 2; IL, interleukin; RAGE, receptors of advanced glycation end products; ROS, reactive oxygen species.

## Discussion

Irrespective of the diabetes status and other underlying comorbidities, blood glucose levels pose a major determining factor in disease severity, response to medical interventions, and mortality among COVID-19 patients.

Vaccines, developed and tested in record time, were approved and authorized for emergency use. Mass immunization programs are rapidly progressing to curb the spread of the virus and reduce disease severity and mortality [120,121]. Although the safety and efficacy of the vaccines were addressed in clinical trials, concerns that the emerging SARS-CoV-2 variants may resist vaccine-induced immunity to varying extent pose a severe threat to the success that the vaccines has so far had in combating this pandemic [122]. However, current evidence show that the Pfizer-BioNTech COVID-19 vaccine (BNT162b2) was effective to a significant extent against both the B.1.1.7 variant (identified in the UK) and the B.1.351 variant (identified in South Africa) [123,124]. Since there is no known cure for COVID-19 and the challenges are overwhelming, it is necessary to identify drugs repurposed to treat COVID-19 [21]. Natural compounds, especially flavonoids, were reviewed for their wide range of anti-inflammatory and immunomodulatory action aganist COVID-19 [125]. In the context of COVID-19, studies have highlighted reduced mortality in patients who had prior use of Metformin [56,126], prompting research groups to investigate Metformin’s effect in nonhospitalized COVID-19 patients to prevent emergencies and disease progression (NCT04510194) [127].

Additionally, clinical studies reported high survival rate/low mortality rate and hospital admissions among outpatient Metformin users [128]. More studies are emerging supporting the beneficial role of Metformin in COVID-19 cases [62,129] (NCT04625985) [129]. In general, the clinical studies on COVID-19 support the multifaceted benefits of Metformin.

However, controversial reports emerge questioning the protective role of ACE2, whether promoting ACE2 expression promotes or protects from SARS-CoV-2 infection as ACE2 is the port of entry for the virus. Hence, the question arises whether Metformin is beneficial and whether the immunomodulatory and glucose regulation of Metformin outweighs the contradiction behind ACE2 phosphorylation [130]. At the cellular level, the steric hindrance hypothesis on blocking the viral entry by Metformin needs further studies to confirm that AMPK-induced Ser 680 phosphorylation of ACE2 in fact causes a conformational change that affects viral–host cell-binding efficacy. Similar studies on blocking phosphorylation of Ser 787 of ACE2 by other drugs prevented the binding of SARS CoV-2 to the receptor by bringing confirmational changes, and this study can be used as a basis for further exploring the effect of ACE2 phosphorylation on viral binding and its implications on host infections and transmissibility of the virus [130].

Although Metformin is an ideal candidate for host-directed therapies, few contraindications were reported in patients. Metformin treatment in COVID-19 patients with diabetes reported hyperglycemia, high lactate dehydrogenase levels, and increased disease severity in a study conducted on 110 COVID-19 patients on Metformin or non-Metformin hypoglycemic drugs [131]. However, another retrospective study revealed no definite association between Metformin and clinical outcomes in COVID-19 patients [132]. This indication of inconsistent results supports the need for further investigations on the effect of Metformin in COVID-19 patients [132].

From the perspective of Metformin’s effect on vaccination, a decreased immune response to trivalent influenza vaccine (TIV) in Metformin-treated type 2 diabetic patients was reported, as indicated by their low antibody response, antibody levels, and low avidity index of influenza-specific IgG antibody [133]. Mechanistic studies revealed the down-regulation of IFN-α expression mediated via mTOR signaling inhibition, where IFN-α plays a vital role in antibody response and protection against viral infection [133]. In this regard, it may also be necessary to study whether Metformin treatment in diabetic individuals has any effect on antibody response and antibody levels in response to the various vaccines that are being currently administered against COVID-19.

Furthermore, Metformin reduces heart failure and inflammation because it reduces metabolic stress by reducing gluconeogenesis and enhanced glucose uptake by peripheral tissue [61,134,135], indicating that Metformin’s benefits outweigh the side effects. Hence, preexisting conditions need to be considered while selecting the proper drug. More clinical studies are warranted to confirm the beneficial role of using Metformin in COVID-19.

Overall, Metformin users showed reduced disease severity, reduced inflammatory response, less likely to need hospital admission, and reduced mortality. In this review, we suggest further exploring the beneficial role of Metformin in viral infections caused by SARS-CoV-2 and the need for investigating and extending its applications in more categories of patients. We explored the molecular mechanism behind the pleotropic actions of Metformin that are linked to a better prognosis in COVID-19 patients with diabetes. In summary, a technically robust and scientifically sound approach is required to investigate the molecular pharmacology of nonclassical targets of Metformin action.

## Conclusions

COVID-19 is a new disease with no known drug for a complete cure. Considering the cost and time toward developing a new drug, repurposing and combinatory drug treatment strategies are efficient options until a specific treatment for COVID-19 is identified. Evidence from recent studies emphasizes the importance of further exploring the Metformin’s role in improving host immune response by potentially targeting specific cell signaling pathways or gut microbiota or energy metabolism. Metformin confers therapeutic benefits in conditions like cancer, cardiovascular complications, polycystic ovary disease, and fatty liver diseases. In the context of SARS-CoV-2 infection, Metformin offers protection not only metabolically but also through the mitigation of complications related to exaggerated immune response and thrombotic events [20]. Although it is safe to use Metformin in COVID-19 with mild to moderate severity, the decision to administer Metformin must be thoroughly examined and judiciously used with conditions such as renal failure and DKA in severely ill COVID-19 patients. Besides, elaborated studies on the protein–protein interaction between host–virus proteins using the currently available cutting-edge technology such as mass spectrometry will help identify precise druggable targets for Metformin intervention. With advanced technologies in molecular biology, genomics, and proteomics, the researchers, scientists, and clinicians will continue to better understand the COVID-19 disease in terms of etiology and pathology of the disease, the possible targets for intervention, drugs that can be used, drug interactions, and the underlying molecular mechanism. However, the challenge remains in bridging the translational gap between research and clinical advancement into patient care and warrants more collaborative research participation between clinicians and academic researchers.
